# A Decade of Change: An Institutional Experience with Breast Surgery in 1995 and 2005

**DOI:** 10.4137/bcbcr.s587

**Published:** 2008-03-25

**Authors:** Amber A. Guth, Beth Ann Shanker, Daniel F. Roses, Deborah Axelrod, Baljit Singh, Hildegard Toth, Richard L. Shapiro, Karen Hiotis, Thomas Diflo, Joan F. Cangiarella

**Affiliations:** 1 Departments of Surgery; 2 Pathology and; 3 Radiology, NYU School of Medicine, Clinical Cancer Center, New York City, NY, U.S.A

**Keywords:** breast cancer, breast surgery, sentinel nodes, breast cancer trends, breast radiology

## Abstract

**Introduction:**

With the adoption of routine screening mammography, breast cancers are being diagnosed at earlier stages, with DCIS now accouting for 22.5% of all newly diagnosed breast cancers. This has been attributed to both increased breast cancer awareness and improvements in breast imaging techniques. How have these changes, including the increased use of image-guided sampling techniques, influenced the clinical practice of breast surgery?

**Methods:**

The institutional pathology database was queried for all breast surgeries, including breast reconstruction, performed in 1995 and 2005. Cosmetic procedures were excluded. The results were analysed utilizing the Chi-square test.

**Results:**

Surgical indications changed during 10-year study period, with an increase in preoperatively diagnosed cancers undergoing definitive surgical management. ADH, and to a lesser extent, ALH, became indications for surgical excision. Fewer surgical biopsies were performed for indeterminate abnormalities on breast imaging, due to the introduction of stereotactic large core biopsy. While the rate of benign breast biopsies remained constant, there was a higher percentage of precancerous and DCIS cases in 2005. The overall rate of mastectomy decreased from 36.8% in 1995 to 14.5% in 2005. With the increase in sentinel node procedures, the rate of ALND dropped from 18.3% to 13.7%. Accompanying the increased recognition of early-stage cancers, the rate of positive ALND also decreased, from 43.3% to 25.0%.

**Conclusions:**

While the rate of benign breast biopsies has remained constant over a recent 10-year period, fewer diagnostic surgical image-guided biopsies were performed in 2005. A greater percentage of patients with breast cancer or preinvasive disease have these diagnoses determined before surgery. More preinvasive and Stage 0 cancers are undergoing surgical management. Earlier stage invasive cancers are being detected, reflected by the lower incidence of axillary nodal metastases.

## Introduction

With the widespread use of breast cancer screening by mammography in the industrialized world, breast cancers are being diagnosed at increasingly earlier stages. In national surveys, approximately 75% of American women 40 years of age and older report having undergone mammography within the last two years (Smigal et al. 2006; Ryerson et al. 2007). Currently ductal carcinoma *in situ* accounts for 22.5% of newly diagnosed breast cancers (Smigal et al. 2006), while in 1990, it accounted for only 2% of all newly diagnosed breast cancers (Adamovich and Simmons, 2003; Esserman, 2006). The American Cancer Society estimates that approximately 212,920 new cases of invasive breast cancer, and 61,980 cases of *in situ* disease would be diagnosed in the United States in the year 2006 (Smigal et al. 2006).

This shift has additionally been attributed to improvements in breast imaging techniques, including the increased use of image-guided sampling techniques, and most recently, the use of magnetic resonance imaging (MRI) evaluation of the breast (Goethem et al. 2006).

In addition, surgical practice has changed dramatically as well over the last decade, particularly with the widespread adoption of the sentinel node technique for axillary staging (Kim et al. 2005), and refinements in breast reconstruction techniques.

How have these changes influenced the clinical practice of breast surgery? We reviewed our institutional experience over a 10 year interval, a period in which both radiologic evaluation and clinical management of breast disease has undergone significant change.

## Methods

The institutional pathology database was queried for all breast surgeries (including breast reconstruction) performed in the years 1995 and 2005. Cosmetic procedures were excluded. The year 1995 was chosen as it was before sentinel node biopsy became the standard of care for axillary staging in early-stage breast cancer, while 2005 was the most recent year for which we had complete data.

The variables examined included year of surgery, patient age, preoperative diagnosis, surgical procedure performed, and pathology results. For patients undergoing simultaneous but unrelated procedures, each procedure was tabulated separately, and the pathology results evaluated separately as well. For patients with invasive ductal carcinoma (IDC) with a component of DCIS, they were tabulated as IDC. Cases of DCIS with microinvasion were included in the DCIS group. The results were tabulated on an Excel spreadsheet, and analyzed utilizing the Chi-square test.

Institutional Review Board approval was granted.

## Results

### Surgical indications

Indications for breast surgery changed significantly over the 10-year study period, with an increased incidence of pre-operatively diagnosed cancers undergoing definitive surgical management, rising from 32.4% in 1995 to 39.8% in 2005. ADH, and to a lesser extent, ALH, became indications for surgical excision. Fewer surgical biopsies were performed for indeterminate abnormalities on breast imaging, decreasing from 34.2% to 16.8%. The results are summarized in Table 1.

There was no significant change in the rate of biopsies performed for a palpable mass, or in the rate of benign biopsies (42.3% vs. 41.1%).

There was no significant change in the percentage of biopsies performed for abnormal or indeterminate findings on fine needle aspiration cytology.

### Surgical procedures

The rate of mastectomy dropped, from 36.8% in 1995 to 14.5% in 2005. Accompanying the increase in sentinel node procedures, the rate of axillary lymph node dissection (ALND) dropped significantly. In 1995, only 6 of 127 patients (4.8%) were evaluated for axillary metastases using the sentinel node technique. By 2005, 246 (64%) of 384 patients were staged solely with sentinel lymphadenectomy. Accompanying the increased recognition of early-stage cancers, the rate of positive axillary staging also decreased significantly, from 43.3% to 25%. Elderly, frail patients, and those with significant life-threatening co-morbidities, did not undergo axillary staging.

In 1995, 60 breast reconstructions were performed. All 20 autologous tissue transfer cases were pedicled transverse rectus abdominis myocutaneous (TRAM) flaps, and the remaining 40 patients underwent implant procedures. In 2005, 35 autologous tissue breast reconstructions were performed (31 TRAM flaps with microvascular reanastomoses and 4 pedicled TRAM flaps) and 80 implant procedures.

### Pathology

There was a higher incidence of DCIS cases in 2005, increasing from 23.4% in 1995 to 32.0%. The incidence of lobular carcinoma in situ (LCIS) was not significantly different in the two index years, probably due to the fact that in the majority of cases, LCIS is still a serendipitous finding, due to its occult nature on both clinical and radiologic evaluation. The incidence of all other pathologic diagnoses was essentially unchanged.

The rate of benign breast biopsies remained constant, with 141 of these lesions being fibroadenomas in 1995 (71%), and 256 fibroadenomas in 2005 (73%).

### Radiology

By 2005, digital mammography was replacing analog mammography. With increasing frequency, Magnetic Resonance Imaging examinations (MRI) were used for high-risk screening and surgical planning, but had not yet been adopted as routine studies. The use of image-guided biopsies changed over the 10-year period (Table 2), with increases in the use of both ultrasound-guided fine needle aspiration (FNA) biopsies and core biopsies. Large-bore Mammotome™ biopsies largely supplanted stereotactic fine-needle aspiration biopsy for the evaluation of mammographic abnormalities.

By 2005, the vast majority of core biopsies were performed under image-guidance by radiologists. The core biopsies performed by the surgeons were either office procedures for diagnosis of a palpable mass, or in women with locally-advanced cancers who were candidates for neoadjuvant therapy, to obtain biomarkers.

Surgeon-operated intraoperative ultrasound has not been routinely used in the institution.

### Faculty

In 1995, three dedicated surgical oncologists and three general surgeons with an interest in breast diseases performed all the breast cases. By 2005, seven dedicated surgical oncologists and one general surgeon managed the breast surgical cases. The volume increase from 1995 to 2005 was due to the expansion of the Breast Service. The case mix remained similar in terms of the percentage of benign disease. The distribution of cancer cases changes however, with an increase in the percentage of early stage breast cancers (i.e. DCIS and node-negative invasive cancers). In both years the surgical philosophy was internally consistent, including the adoption of sentinel node techniques, the role of re-excision following breast-conserving surgery, the use of skin-sparing mastectomy, and the reluctance to perform nipple/areolar-sparing mastectomy. In 2005, the use of preoperative MRI varied among the surgical oncologists, particularly as MRI-guided core biopsies and preoperative needle-localization techniques were not available within the institution at that time.

In 2003, the Pathology Department created a Division of Breast Pathology with a core group of dedicated breast pathologists. Throughout the study period, there has been a dedicated Division of Breast Imaging, staffed only by dedicated mammographers.

## Discussion

With increased awareness and availability of routine breast cancer screening, breast cancers are being diagnosed at earlier stages. Currently, over 70% of all newly diagnosed breast cancers in the United States are Stages 0–2 (van de Vijver, 2005; Smigal et al. 2006).

Accompanying this are major technologic changes in breast imaging. Digital mammography is quickly becoming the standard of care, especially in the evaluation of premenopausal women (Pisano et al. 2005). When compared to film mammography, digital mammography has been found to be more accurate in women under the age of 50 years, in women with radiologically dense breasts, and in pre/perimenopausal women.

The widespread adoption of large-bore needle image-guided biopsy techniques has had a major impact on how breast cancers are diagnosed. Rather than undergoing an initial diagnostic surgical procedure, increasingly patients go to the operating room with a histologic diagnosis of a high-risk or malignant lesion (Liberman, 1999; Crowe et al. 2002). Crowe et al. (10) reported on the experience at the Cleveland Clinic from 1995 to 2000. During that time, the percentage of diagnostic breast biopsies performed as image-guided core biopsies rather than surgical biopsies increased from 31% to 68%. A similar change is reflected in our data (Tables 1&2).

Within the last few years the role of breast MRI has become better defined. In particular, it is now being used for surgical planning in the setting of a known breast cancer, both to evaluate the extent of known disease, and for the detection of multifocal, multicentric, and contralateral malignancies (Goethem et al. 2006) (Lehman et al. 2007). It appears that tumor diameter measured on MRI correlates more closely with the histologic measurements, compared to the diameters measured on mammography and ultrasound. Thus, it is being increasingly used in planning for breast-conserving therapy.

### Indications for surgery

Over this ten-year period the indications for breast surgery changed, shifting away from diagnostic procedures to therapeutic interventions. This is reflected by the decreased rate of procedures being performed for abnormal mammographic findings, from 32.4% in 1995 to 16.8% in 2005, and the increase in preoperative diagnoses of breast cancer or pre-malignant lesions such as ADH/ALH from 32.4% in 1995 to 43.1% in 2005.

### Surgical procedures

From 1995 to 2005, the mastectomy rate in our institution decreased by more than 50%. This is due to the liberalization of indications for breast conserving therapy to include synchronous tumors in same quadrant and lobular cancers, the diagnosis of earlier stage cancers, as well as growing interest in oncoplastic techniques for breast reconstruction following partial mastectomy. These new techniques strive to optimize the efficacy of breast conserving therapy (BCT) with respect to both appropriate local control, as well as cosmetic results (Masetti et al. 2006). The increase in BCT may also be influenced by the use of preoperative MRI—perhaps patients (and surgeons) are more comfortable with the decision to proceed with breast conservation when an MRI has revealed no additional suspicious lesions in either breast.

The increase in sentinel node procedures reflects that this technique has supplanted the diagnostic axillary dissection for routine staging of the axilla in early stage breast cancer.

### Pathology

Interestingly, the rate of benign biopsies was similar in both years. The preoperative evaluation, and indications for surgical removal of presumed benign lesions in young women, did not change appreciably during the study period, and this may explain the constant rate of benign biopsy.

When considering the ADH/ALH group, the final surgical pathology results maybe somewhat misleading for this group. With the increase in image-guided diagnostic core biopsies, we are seeing more cases of minimal volume disease in which all involved tissue has been removed by the large bore vacuum-assisted biopsies, with no residual atypia on the subsequent surgical excision. This may explain the decrease in rate of ADH/ALH in surgical specimens in 2005 (personal communication, Baljit Singh).

### Conclusions

The surgical management of breast disease has changed significantly in our institution during the last decade, reflecting the widespread refinements in breast cancer diagnosis and management. The management of breast disease has shifted from generalists to dedicated surgical oncologists and breast pathologists.

While the rate of benign breast biopsies has remained constant, more pre-invasive lesions and Stage 0 cancers are undergoing surgical management. A greater percentage of patients with breast disease have their diagnoses determined before surgery. Earlier stage invasive cancers are being detected, reflected by the lower incidence of axillary nodal metastases. Sentinel node biopsy has supplanted diagnostic axillary dissection Correspondingly there has been a greater shift towards breast conserving therapy.

## Figures and Tables

**Table 1.** Comparison of surgical experience: 1995 and 2005.

**Characteristics**	**1995 (n = 661)**	**2005 (n = 1005)**	**p-values**
***Indications for surgery***			
Cancer	214(32.4%)	394(39.2%)	<0.001
Abnormal x-ray	215(32.4%)	169(16.8%)	<0.001
Palpable mass	199(30.1%)	342(34.0%)	NS
Abnormal FNA	30(4.5%)	61(6.1%)	NS
ADH	0(0%)	31(3.1%)	<0.001
ALH	0(0%)	8(0.8%)	<0.025
Mean age (years)	55 +/− 14	53 +/− 15	NS
***Type of surgery***	*Note: 27 patients underwent multiple procedures*		
Mastectomy	242(36.6%)	150(14.9%)	<0.001
Excisional biopsy	237(35.9%)	191(19.0%)	<0.001
Needle-localization biopsy	58(8.7%)	235(23.4%)	<0.001
Partial mastectomy	151(22.3%)	424(42.2%)	<0.001
Core biopsy by surgeon	23(3.5%)	3(0.3%)	<0.001
***Axillary staging***			
Sentinel node*	6(1%)	246(24.5%)	<0.001
Axillary dissection**	121(18.3%)	138(13.7%)	<0.025
***Pathology****(Included multiple diagnoses if significant)*			
Benign	280(42.3%)	413(41.1%)	NS
ADH/ALH	94(19.2%)	138(13.7%)	NS
DCIS	155(23.4%)	322(32.0%)	<0.001
LCIS	34(5.1%)	75(7.4%)	NS
IDC	130(19.7%)	238(23.7%)	NS
ILC	24(3.6%)	43(4.3%)	NS
IDLC	3(0.5%)	11(1.1%)	NS
# positive/all axillary staging	54/127(43.3%)	96/384(25.0%)	<0.001

* Performed as initial diagnostic staging technique.

** Performed for suspected/known axillary metastases, or following positive sentinel node biopsy.

**Table 2.** Comparison of NYU Image-Guided Needle Biopsies: 1995 and 2005.

**Biopsy method (# of procedures)**	**1995**	**2005**
Ultrasound-guided FNA and core biopsies	1	851
Mammographic-guided FNA and mammotome biopsies	36	199
Total # of procedures for year	37	1050
